# Heterogeneity of Phosphatidylinositol-3-Kinase (PI3K)/AKT/Mammalian Target of Rapamycin Activation in Cancer: Is PI3K Isoform Specificity Important?

**DOI:** 10.3389/fonc.2017.00330

**Published:** 2018-01-22

**Authors:** Célia Cintas, Julie Guillermet-Guibert

**Affiliations:** ^1^INSERM U1037, CRCT, Université Toulouse III Paul Sabatier, Toulouse, France; ^2^Laboratoire d’Excellence TouCAN, Toulouse, France

**Keywords:** phosphatidylinositol-3-kinase pathway, proteomics, transcriptomics, genomics, data integration, precision medicine, predictor of therapeutic response, isoform specificity

## Introduction

**Figure 1:**
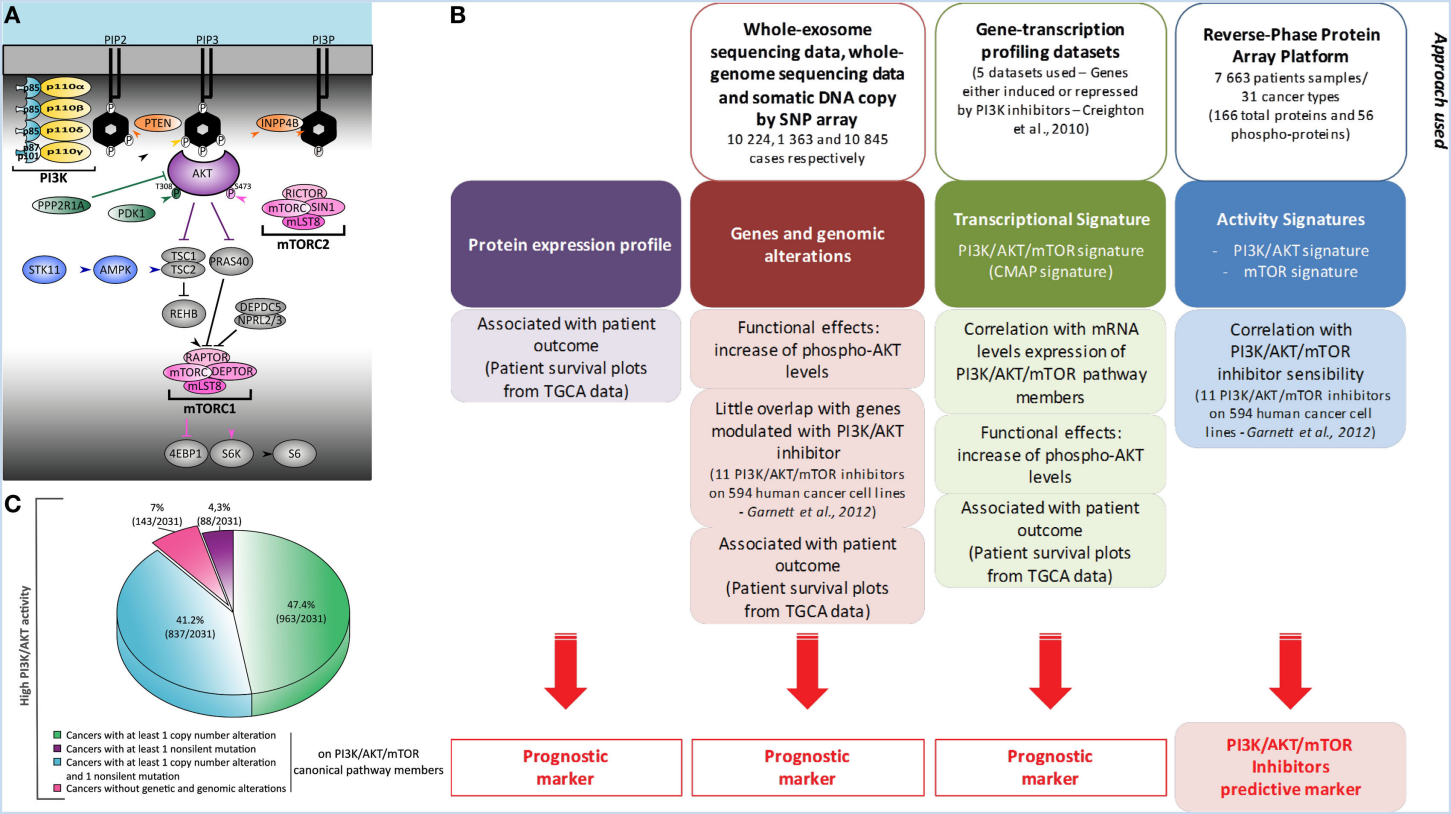
Heterogeneity of phosphatidylinositol-3-kinase (PI3K)/AKT/mammalian target of rapamycin (mTOR) activation in cancer: global and comprehensive mapping by a multiscale integrated approach. **(A)** Representation of PI3K/AKT/mTOR canonic members. Most omics results use data obtained under treatment with pan-PI3K inhibitors which still display relative isoform specificity, or with PI3Kα-selective inhibitors associated with genetic alterations. Usually, only the expression of *PIK3CA* and *PIK3CB* is studied. Production of PIP_3_ at the plasma membrane is, however, performed by four enzymes: PI3Kα, PI3Kβ, PI3Kδ, and PI3Kγ. They are composed of a regulatory subunit (p85 or p101/p87) and a catalytic subunit (p110α, p110β, p110δ, or p110γ). **(B)** This schematic summarizes the bioinformatic meta-analysis performed in the study by Zhang et al. (2) using two PI3K/AKT/mTOR transcriptional signatures of Creighton et al. and Garnett et al. (3, 4). **(C)** Representation of the molecular alterations found in cancer patients with high PI3K/AKT score. Molecular alterations in patients with reverse-phase protein array (RPPA) score values ≥0.5 are shown. These RPPA scores were normalized to SDs from the median across all cancers.

One of the most common events in human cancer is hyperactivation of the phosphatidylinositol-3-kinase (PI3K)/AKT/mammalian target of rapamycin (mTOR) signaling pathway, generally described as a consequence of genetic alterations of pathway members. The core components of the pathway are depicted in Figure 1A. There are four members of the class I PI3Ks, which act upstream of this pathway, performing the conversion of phosphatidylinositol-4,5-bisphosphate (PIP_2_) into phosphatidylinositol-3,4,5-trisphosphate (PIP_3_). This lipid produced in the inner leaflet of the plasma membrane controls a range of cellular actions including cell growth, migration, metabolism, survival and proliferation. Class I PI3K activity in vertebrates regulates both physiological and pathological processes (1).

Phosphatidylinositol-3-kinase activity has been implicated in a variety of different cancers, hence this class of enzymes is a prime drug target for anticancer therapies (5). However, initial phase I/II clinical trials of small molecule PI3K inhibitors show that the predictive markers of efficiency of these drugs need to be improved. The presence of *PIK3CA* mutation in the primary tumor alone is not a sufficient predictive marker of efficiency (6, 7). Signal-targeted therapy would benefit from the identification of patients more likely to respond.

## A Recent Multiscale Omics Approach Mapped PI3K/AKT/mTOR Activation in Cancer

Zhang et al. analyzed in an unbiased fashion both known molecular mechanisms by which the PI3K/AKT/mTOR pathway is upregulated in human cancers, as well as other possibly unrelated genetic alterations (2). They examined The Cancer Genome Atlas open access omics data (including genomic mutations by whole-genome sequencing, gene copy number by single-nucleotide polymorphism array, or RNA expression by whole-exome sequencing) across 11,219 human cancers representing 32 distinct major types. The authors also used reverse-phase protein array (RPPA) analysis to assess the level of expression of 166 total proteins and 56 phosphorylated proteins (Figure 1B). Phosphoproteome-based PI3K/AKT and mTOR activity signatures (p-AKT_S473/T308_, p-GSK3_S9_, p-PRAS40_T246_, p-TSC2_T1462_ and p-mTOR_S2448_, p-RICTOR_T1135_, p-4EBP1_S65/T34/T46/T70_, p-S6K_T389_, p-S6_S235/S236/S240/S244_, respectively) by RPPA analysis were found to be correlated. Transcriptomics analysis found that the levels of expression of a selected list of members of the PI3K/AKT pathways were not correlated with the activation of PI3K or mTOR signaling nodes by RPPA. However, the authors confirmed a correlation between the transcriptional expression and alterations in the copy number of the so-called core genes. All mutated residues of members of the PI3K/AKT/mTOR pathway resulted in the expected increase in activity of the PI3K/AKT/mTOR pathway. Therefore, modifications to DNA, mRNA expression, and phosphoprotein levels were found to be functionally relevant in the hyperactivation of the PI3K pathway associated with cancers.

## Not all Cases of Increased PI3K/AKT/mTOR Pathway Activity Can be Explained by the Canonic Genetic Alterations Associated with PI3K Signaling

In most scenarios, increased AKT activity can be explained by genetic or genomic alterations to members of the PI3K/AKT/mTOR pathway; however, this is not the case for all instances of AKT hyperactivation (Figure 1C). In 764 of 7,099 tumors, including mostly lower grade glioma, pheochromocytoma and paraganglioma, prostate adenocarcinoma, and kidney renal clear cell carcinoma, the level of phospho-AKT was increased without any of the genetic or genomic alterations described as being functionally coupled to this pathway. In addition, upregulation of mTOR pathway activity is also associated with non-canonical alterations, such as *IDH1* (Isocitrate dehydrogenase 1) or *VHL* (Von Hippel-Lindau syndrome) mutations, miRNA modulation, and ERK, SRC, and NDRG1 activation. Further work is required to understand the complex mechanisms involved in upregulation of the PI3K/AKT/mTOR pathway by non-canonical alterations.

## Cellular Activity of the PI3K/AKT/mTOR Pathway is a Predictive Marker of Sensitivity to PI3K/mTOR Inhibitors

Analysis of knock-down (shRNAs) data for pathway effectors (3, 4) and a correlation matrix of PI3K/AKT/mTOR, MYC and active KRAS gene signatures, showed a convergence of MYC and KRAS oncogenic signaling pathways with the PI3K/AKT/mTOR transcriptomic signature. However, the PI3K/AKT/mTOR transcriptomic signature was not a predictive marker of PI3K/mTOR inhibitor efficacy, but was associated with patient outcome/survival. Instead, a list of 146 genes significantly associated with tumor cell sensitivity to pathway inhibitors correlated with the PI3K/AKT phosphoproteomic signature. Therefore, functional assessment of the PI3K pathway was linked with the sensitivity to its inhibitors, while its final transcriptomic targets were only indicative of the impact of this pathway in the malignancy of each cancer (Figure 1B).

## Both Pan-PI3K and Isoform-Selective Compounds are Progressing in the Clinic

There are four members of the class I PI3Ks: PI3Kα, PI3Kβ, PI3Kγ, and PI3Kδ, and these have different functions in physiopathology (1). PI3Kα and PI3Kβ are ubiquitously expressed, whereas PI3Kγ and PI3Kδ are preferentially expressed in leukocytes or the vascular system, but are also overexpressed in some primary tumors. This led to the concept that isoform-specific functions of PI3K enzymes in non-transformed cells become redundant in transformed cells due to a possible dysregulation of upstream signaling. However, several research teams, including us, show that certain cancers are solely dependent on one isoform of PI3K. For example, a prostate cancer model induced by inactivation of the tumor suppressor gene *PTEN* (a phosphatase that negatively regulates the PI3K/AKT signaling pathway—see Figure 1A) depends only on the PI3K activity of PI3Kβ. In addition, thyroid tumors induced by inactivation of *PTEN* depend only on PI3Kα activity, suggesting a tissue-context phenomenon (8, 9). Selective engagement by receptor tyrosine kinases (RTKs) or G-protein-coupled receptors (GPCRs) to specific PI3K isoforms when all the RTK-linked (α, β, and δ) PI3Ks or all the GPCR-linked PI3Ks (β and γ) are expressed in a given cell type remains to be demonstrated (Figure 1A). Indeed, the two GPCR-activated class I PI3Ks were found to be redundant in various cell systems (10). In the context of tumor initiation, evidence clearly shows isoform specificity (11, 12). In neoplastic cells harboring high levels of genomic instability, isoform selectivity could be transient, because, once specific isoforms are inhibited, other class I enzymes can become activated by other mechanisms (13, 14). For example, inhibition of PI3Kβ relieves feedback inhibition of RTKs, thus reactivating PI3Kα (14). Conversely, inhibition of PI3Kα (in tumors harboring RTK activation or an activating oncogenic mutation in *PIK3CA*) leads to the activation of PI3Kβ by inactivation of the tumor suppressor *PTEN* (15). With limited success in patients receiving dose-limiting toxicity amounts of pan-PI3K inhibitors, literature suggests that a selective inhibition of one isoform could lead to a better efficacy/toxicity ratio. Novel, isoform-selective inhibitors are currently progressing quickly in the clinic (16, 17). Moreover, isoform-sparing compounds have been successfully developed to target the oncogenic driving PI3K and the immune restricted PI3Ks, in particular in the highly inflammatory triple-negative breast cancer setting (18). These isoform-selective or isoform-sparing inhibitors still have serious toxicity issues, as seen with the FDA-approved idelalisib. The main resistance mechanism of pan-class I PI3K inhibitors is *via* reactivation of the MAPK pathway. However, most patients treated with the pan-PI3K inhibitor buparlisib (BKM120) and the MEK1/2 inhibitor trametinib (GSK1120212) experienced severe grade 3/4 adverse events, and 31% of them arrested their treatment (19). On-target toxicity is also major issue for PI3K inhibitors, hence more balanced pan-PI3K, pan-PI3K/mTOR, or dual isoform PI3K inhibitors are being developed.

Many questions need to be answered regarding clinically relevant PI3K inhibition in cancer. This inhibition can be either isoform-specific or pan-PI3K, the later possibly with a selective inhibition efficiency for each isoform. Some of the more pertinent questions are outlined below:

i- While it was obvious to hit PI3Kδ in hematological malignancies due to the overexpression of this isoform in this cell type, how can we predict the sensitivity to isoform-specific drugs for the two ubiquitously expressed yet critical for cancerogenesis PI3Ks, PI3Kα, and PI3Kβ?
ii- Should we take into account the activity of stromal PI3Kγ and PI3Kδ in solid cancers?
iii- Are inter-isoform compensatory mechanisms similar for all types of cancers?
iv- How can we predict which other isoforms are involved in resistance mechanisms?

It thus remains critical to delineate for each type of cancer and ultimately each individual patient which PI3K isoform(s) to target, and the level of inhibition at which each of these isoforms needs to be targeted so as to reach an optimal cost benefit ratio.

## Conclusion/Perspectives

Zhang et al. assessed PI3K/AKT and mTOR activity signatures, the PI3K/AKT/mTOR transcriptional signature and genetic and genomic alterations of canonical members of the pathways, to strongly demonstrate the major contribution of PI3K/AKT/mTOR pathway deregulation to poor survival in different types of cancer. Direct mutation of genes of the pathway is not the only common mechanism of activation of this pathway in cancer cells. It is also necessary to consider other non-canonical members of the PI3K/AKT/mTOR pathway to evaluate its activity, as well as other apparently unrelated genetic or genomic alterations. Such examples of meta-analysis largely pave the way to a better understanding of PI3K oncogenic signaling regulation and interconnection with its transcriptional targets in cancers. Finally, the first specific PI3K inhibitor to be approved for clinical use is a PI3Kδ-selective inhibitor, which has shown high efficacy in phase III clinical trials for B cell lymphoma (20). Evidence for PI3K isoform selectivity in tumors should not be neglected in these large-scale omics studies. An analysis of PI3K signaling taking into account its entire complexity (isoform selectivity) is important in order to establish optimal antitumoral therapies and select patients likely to benefit from PI3K-targeted therapies.

## Author Contributions

All authors listed have made a substantial, direct, and intellectual contribution to the work and approved it for publication.

## Conflict of Interest Statement

The authors declare that the research was conducted in the absence of any commercial or financial relationships that could be construed as a potential conflict of interest.
